# RETRACTED: Park et al. *Sargassum serratifolium* Extract Attenuates Interleukin-1β-Induced Oxidative Stress and Inflammatory Response in Chondrocytes by Suppressing the Activation of NF-κB, p38 MAPK, and PI3K/Akt. *Int. J. Mol. Sci.* 2018, *19*, 2308

**DOI:** 10.3390/ijms27135928

**Published:** 2026-07-01

**Authors:** Cheol Park, Jin-Woo Jeong, Dae-Sung Lee, Mi-Jin Yim, Jeong Min Lee, Min Ho Han, Suhkmann Kim, Heui-Soo Kim, Gi-Young Kim, Eui Kyun Park, You-Jin Jeon, Hee-Jae Cha, Yung Hyun Choi

**Affiliations:** 1 Department of Molecular Biology, College of Natural Sciences, Dong-eui University, Busan 47340, Republic of Korea; parkch@deu.ac.kr; 2 Freshwater Bioresources Utilization Bureau, Nakdonggang National Institute of Biological Resources, Sangju 37242, Republic of Korea; jwjeong@nnibr.re.kr; 3 National Marine Biodiversity Institute of Korea, Seocheon 33662, Republic of Korea; daesung@mabik.re.kr (D.-S.L.); mjyim@mabik.re.kr (M.-J.Y.); lshjm@mabik.re.kr (J.M.L.); mhhan@mabik.re.kr (M.H.H.); 4 Department of Chemistry, College of Natural Sciences, Center for Proteome Biophysics and Chemistry Institute for Functional Materials, Pusan National University, Busan 46241, Republic of Korea; suhkmann@pusan.ac.kr; 5 Department of Biological Sciences, College of Natural Sciences, Pusan National University, Busan 46241, Republic of Korea; khs307@pusan.ac.kr; 6 Department of Marine Life Sciences, School of Marine Biomedical Sciences, Jeju National University, Jeju 63243, Republic of Korea; immunkim@jejunu.ac.kr (G.-Y.K.); youjinj@jejunu.ac.kr (Y.-J.J.); 7 Department of Oral Pathology and Regenerative Medicine, School of Dentistry, Institute for Hard Tissue and Biotooth Regeneration, Kyungpook National University, Daegu 41940, Republic of Korea; epark@knu.ac.kr; 8 Department of Parasitology and Genetics, College of Medicine, Kosin University, Busan 49267, Republic of Korea; 9 Anti-Aging Research Center and Blue-Bio Industry RIC, Dong-eui University, Busan 47227, Republic of Korea; 10 Department of Biochemistry, College of Korean Medicine, Dong-eui University, Busan 47227, Republic of Korea

The journal retracts the article titled “*Sargassum serratifolium* Extract Attenuates Interleukin-1β-Induced Oxidative Stress and Inflammatory Response in Chondrocytes by Suppressing the Activation of NF-κB, p38 MAPK, and PI3K/Akt” [1], cited above. 

Following publication, concerns were brought to the attention of the Editorial Office regarding the integrity of several figures published in this article [1].

In accordance with standard journal procedures, the Editorial Office and Editorial Board conducted an investigation that identified indications of inappropriate editing and partial duplication within images presented in Figures 2c, 3b and 5b of this article [1]. Although the authors cooperated during the investigation, the explanations and supporting materials provided were not deemed sufficient by the Editorial Board to resolve the identified integrity issues and the provenance of the images.

Consequently, the Editorial Board has lost confidence in the reliability of the findings and decided to retract this publication [1], as per MDPI’s retraction policy. 

This retraction was approved by the Editor-in-Chief of the journal *IJMS*. 

The authors do not agree on this decision.
